# Spectral Reflectance Estimation from Camera Response Using Local Optimal Dataset and Neural Networks

**DOI:** 10.3390/jimaging10090222

**Published:** 2024-09-09

**Authors:** Shoji Tominaga, Hideaki Sakai

**Affiliations:** 1 Department of Computer Science, Norwegian University of Science and Technology, 2815 Gjøvik, Norway; 2 Department of Business and Informatics, Nagano University, Ueda 386-1298, Japan; 3 Professor Emeritus, Kyoto University, Kyoto 606-8501, Japan; hsakai@i.kyoto-u.ac.jp

**Keywords:** surface-spectral reflectance, reflectance estimation, multispectral imaging, local optimal dataset, neural network, training-based approach, model-based approach

## Abstract

In this study, a novel method is proposed to estimate surface-spectral reflectance from camera responses that combine model-based and training-based approaches. An imaging system is modeled using the spectral sensitivity functions of an RGB camera, spectral power distributions of multiple light sources, unknown surface-spectral reflectance, additive noise, and a gain parameter. The estimation procedure comprises two main stages: (1) selecting the local optimal reflectance dataset from a reflectance database and (2) determining the best estimate by applying a neural network to the local optimal dataset only. In stage (1), the camera responses are predicted for the respective reflectances in the database, and the optimal candidates are selected in the order of lowest prediction error. In stage (2), most reflectance training data are obtained by a convex linear combination of local optimal data using weighting coefficients based on random numbers. A feed-forward neural network with one hidden layer is used to map the observation space onto the spectral reflectance space. In addition, the reflectance estimation is repeated by generating multiple sets of random numbers, and the median of a set of estimated reflectances is determined as the final estimate of the reflectance. Experimental results show that the estimation accuracies exceed those of other methods.

## 1. Introduction

Knowledge of the surface-spectral reflectances of objects is essential in fields such as color science, image science and technology, computer vision, and computer graphics. Therefore, issues in estimating the surface-spectral reflectances from camera responses have been studied alongside the development of cameras and imaging systems, leading to the proposal of numerous methods. Methods used to estimate the surface-spectral reflectances based on camera responses can be classified into two primary approaches: model-based approach [1,2,3,4,5,6,7,8,9,10,11,12,13,14] and training (or learning)-based approach [15,16,17,18,19,20,21,22,23,24,25,26,27,28,29,30,31,32,33,34,35,36,37].

In the model-based approach, the camera responses are described using camera spectral sensitivities, surface-spectral reflectance, and illuminant spectral power distributions. This is the traditional and more commonly used approach and includes finite-dimensional modeling methods [1,3] and Wiener estimation methods [4,5,6,7,8,9,10,11,12]. Wiener estimation methods are based on a statistical approach in which noise in the imaging system and a certain spectral reflectance statistic are considered. Linear minimum mean square error (LMMSE) [13] is an improved Wiener estimation method. Recently, a method [14] was proposed to estimate the surface-spectral reflectances from camera responses using a local optimal reflectance dataset, in which a spectral reflectance database was utilized to locally determine the candidates to optimally estimate the spectral reflectance. The best spectral reflectance was effectively estimated using only the local optimal dataset without using the entire spectral reflectance database.

The training-based approach is typically constructed without knowledge of the camera spectral sensitivities and illuminant spectral distributions. Rather, it uses a large training dataset, which is a large table comprising a pair of camera responses and the corresponding spectral reflectances. Regression methods directly establish the relationship between RGB responses and spectral reflectances and include support vector regression [19,20], kernel regression [21,22], and linear regression [23,24].

In recent years, neural networks have been used in reflectance estimation problems in many areas. In fact, material reflectance or albedo was estimated based on a neural network approach [28,29,30,31]. A neural network approach was proposed for leaf chlorophyll and carotenoid estimation using hyperspectral reflectance [32]. Soil organic carbon was predicted using VNIR spectroscopy employing neural network modeling [33]. Material type recognition was considered based on IR reflectances and color images [34].

Regarding spectral reflectance estimation, the use of neural networks has been considered to construct a map between the low-dimensional color signal space and higher-dimensional spectral space [35,36,37], where a neural network model optimized to predict color reflectance for multiple coating products was demonstrated in [35], and a map between the CMYK color space and the spectral space using neural networks was proposed in [36]. In [37], a neural network method to estimate spectral reflectance was applied to a dual imaging system with a color projector and color camera, where mapping was constructed between six-dimensional color signals and the spectral space. The neural network was then trained using numerous samples with known spectral reflectance, including the Munsell dataset and the 24 color checker dataset.

In this study, we propose a novel method that combines model- and training-based approaches to improve the estimation accuracy of spectral reflectance from image data. The proposed method comprises two stages. The first stage is based on the model base, where the local optimal reflectance dataset is selected as a set of the most reliable candidates for reflectance estimation from a standard reflectance database. The second stage employs a training-based method, where the best estimate is determined by applying a neural network method to the selected local optimal dataset only. Our imaging system is a multispectral image acquisition system extended from a simple RGB system, in which an RGB camera captures multiple images of an object scene under multiple light sources with different illuminant spectra in the visible range.

In the following, Section 2 describes the observation model for an image-acquisition system that uses an RGB camera and multiple light sources. We adopt a general model in which the camera responses are described by combining camera spectral sensitivities, illuminant spectral power distributions, unknown surface-spectral reflectance, additive noise terms, and a gain parameter.

Section 3 describes the development of the proposed spectral estimation method. First, we describe the selection of the local optimal reflectance dataset. The actual camera responses for the target object are compared with the observations predicted from the respective spectral reflectances in the reflectance database. Prediction errors are calculated for all reflectances in the database, and the local optimal candidates for reflectance estimation are selected in the order of the lowest prediction error. Second, we determine the best reflectance using a neural network based only on a locally optimal dataset. A random convex linear combination of the local optimal dataset becomes the training data of reflectance for the neural network, and the network is trained to minimize the mean square error (MSE). An additional procedure is presented to obtain reliable reflectance estimates.

Section 4 presents the experiments performed to validate the proposed methods for estimating the surface spectral reflectances. Various mobile phone cameras, LED light sources, a standard spectral reflectance database, and standard test samples are used in these experiments. The performance of the proposed method is examined in detail and compared with that of other methods.

Section 5 discusses the relationship between the statistics of the random numbers used and estimation accuracy.

## 2. Observation Model

**Figure 1:**
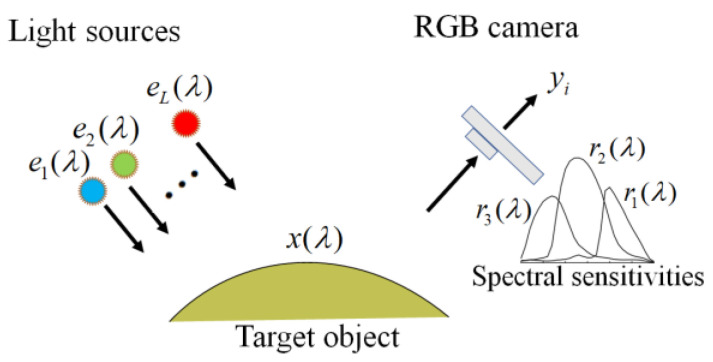
Conceptual diagram of our image acquisition system [14].

The observation model of our image-acquisition system is shown in Figure 1 (see [14]). It was constructed using an RGB camera with three color channels (*c* = 1, 2, 3) and multiple light sources with *L* different illuminant spectra (*l* = 1, 2,..., *L*). Hence, we obtained *m* = 3*L* observations for a single target object. The observation $y_{i}$ of the camera outputs is expressed as follows:

(1) $$y_{i}\;=\;g\int_{400}^{700}x(\lambda )e_{l}(\lambda )r_{c}(\lambda )d\lambda \;\;+\;n_{i},\;\;\;\;\;\;\;\;\;\;\;\;\;\;\;\;\;\;\;\;\;\;\;\;\;\;\;\;\;\;\;\;\;\;\;\;\;\;\;\;\;\;\;\;\;\;\;\;\;\;\;\;\;\;\;\;\;\;\;\;\;\;\;\;\;\;\;\;\;\;\;\;\;\;\;\;\;\;\;\;\;\;\;\;\;\;\;\;\;\;\;\;\left(i=1,\;2,\ldots ,m\right),$$

where x(λ) is the surface-spectral reflectance of the target object, $e_{l}(\lambda )$ (*l* = 1, 2, …, *L*) represent the spectral power distribution of the light sources, $r_{c}(\lambda )$ (*c* = 1, 2, 3) denote the spectral sensitivity functions of the camera. The wavelength λ is in the visible range of 400–700 nm. The additive noise $n_{i}$ in the imaging system is assumed to be white noise with zero mean and variance *a* and is uncorrelated with x(λ). Here, $y_{i}\;$ represent the digital camera outputs, while x(λ), $e_{l}(\lambda )$, and $r_{c}(\lambda )$ are physical quantities. The coefficient g in Equation (1) is a gain parameter used to convert the model outputs to the practical digital output. The parameter g is unique to the imaging system and depends on the conditions of the imaging system, such as the locations of the camera and light sources, including illumination intensities. How to determine the noise variance *a* and the gain parameter *g* was shown in [13].

The spectral functions of reflectance, illuminants, and sensitivities are sampled at *n* wavelength points with equal intervals in the range of 400–700 nm and described using *n*-dimensional column vectors as follows:

(2) $$x=\left[\begin{array}{c} x(\lambda_{1})\\ x(\lambda_{2})\\ \vdots \\ x(\lambda_{n}) \end{array}\right],\;\;\;\;\;\;e_{l}=\left[\begin{array}{c} e_{l}(\lambda_{1})\\ e_{l}(\lambda_{2})\\ \vdots \\ e_{l}(\lambda_{n}) \end{array}\right],\;\;\;\;\;\;r_{c}=\left[\begin{array}{c} r_{c}(\lambda_{1})\\ r_{c}(\lambda_{2})\\ \vdots \\ r_{c}(\lambda_{n}) \end{array}\right],$$

where *i* = 1, 2, …, *L* and *c* = 1, 2, 3. The discrete representation of the observation model is expressed as

(3) y = gAx + n,

where

(4) $$y=\;\left[\begin{array}{c} y_{1}\\ y_{2}\\ \vdots \\ y_{m} \end{array}\right],\;\;\;\;A=\;\left[\begin{array}{c} {\left(e_{1}.*r_{1}\right)}^{t}\Delta \lambda \\ {\left(e_{2}.*r_{2}\right)}^{t}\Delta \lambda \\ \vdots \\ {\left(e_{L}.*r_{3}\right)}^{t}\Delta \lambda \end{array}\right],\;\;\;\;n\;=\;\left[\begin{array}{c} n_{1}\\ n_{2}\\ \vdots \\ n_{m} \end{array}\right]$$

The symbol (.*), superscript *t*, and Δλ represent element-wise multiplication, matrix transposition, and the wavelength sampling interval, respectively. Therefore, **A** is an (*m × n*) matrix defined by the illuminant spectra and spectral sensitivities, and ***n*** is an *n*-dimensional noise vector.

## 3. Reflectance Estimation Method

### 3.1. Selection of Local Optimal Reflectance Dataset

**Figure 2:**
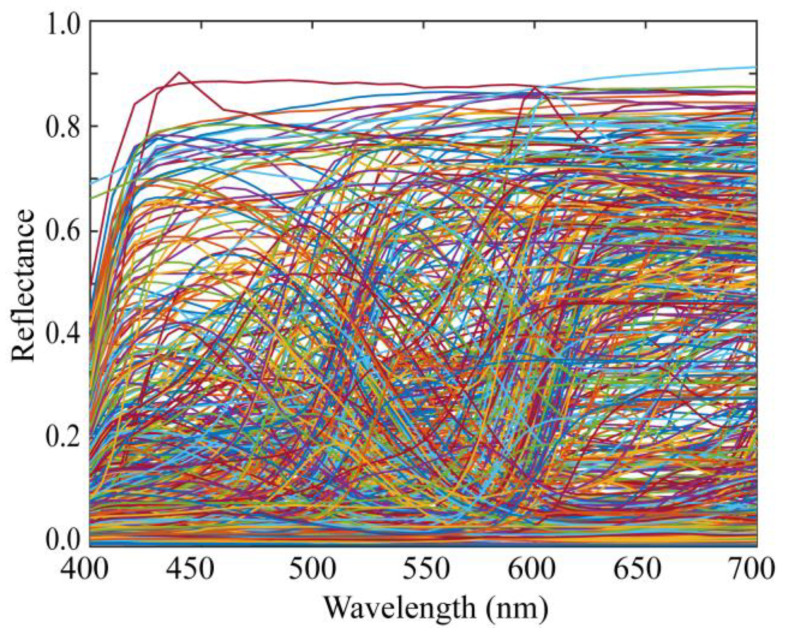
Database of surface-spectral reflectance.

Figure 2 shows the standard database of surface-spectral reflectance used in this study, which comprises Dupont spectral data, Munsell spectral data, and various object spectral data, including manmade objects such as papers, paints, and plastics, as well as natural objects such as rocks, leaves, skins, oranges, and apples. This database is available at http://ohlab.kic.ac.jp/ (accessed on 1 July 2024), which is a dataset of 1776 spectral reflectances. Let $N_{D}$ (1776) be the number of spectral reflectances in a database. All spectral curves are sampled at 61 (=*n*) points with 5 nm intervals in the visible range of 400–700 nm and represented by 61-dimensional column vectors $x_{i}$ (*i* = 1, 2,..., $N_{D}$).

First, the observations are predicted using Equation (3) as an $gAx_{i}$ for each spectral reflectance $x_{i}$ in the database. The prediction error for observation **y** is then calculated as follows:

(5) $$L_{i}={\left\|y-gAx_{i}\right\|}_{2}^{2}\;\;(i=1,\;2,\ldots ,\;N_{D}),$$

where norm ${\left\|•\right\|}_{2}^{2}$ is defined as ${\left\|z\right\|}_{2}^{2}=z_{1}^{2}+z_{2}^{2}+\ldots +z_{m}^{2}$. Secondly, the prediction errors are arranged in ascending order as $L_{\left(1\right)}\leq L_{\left(2\right)}\leq \cdots \leq L_{\left(N_{D}\right)}$, and the corresponding spectral reflectances are $x_{\left(1\right)}$, $x_{\left(2\right)}$, …,$x_{\left(N_{D}\right)}$. Finally, the first K spectral reflectances,$x_{\left(1\right)}$, $x_{\left(2\right)}$, …,$x_{\left(K\right)}$ are selected as local optimal candidates to estimate the spectral reflectance. 

### 3.2. Determination of Reflectance Estimate Using Neural Network

The best estimate is determined using a neural network based only on the local optimal dataset $\left(x_{\left(1\right)},\;x_{\left(2\right)},\;\ldots ,\;x_{\left(K\right)}\right)$.

#### 3.2.1. Making the Training Data

The training data are a large table comprising a pair of spectral reflectances and corresponding observations. The training data for the spectral reflectance are composed of the original local optimal dataset obtained in Section 3.1 and the augmented data made by the convex linear combination of the local optimal dataset. Let $N_{T}$ be the number of training data and ${\hat{x}}_{i}$ (*i* = 1,2,…, $N_{T}$) be the spectral reflectance used for training. The spectral reflectances are described as

(6) $$\begin{array}{l} {\hat{x}}_{i}\;=\;x_{\left(i\right)},\;\;\;\;\;\;\;\;\;\;\;\;\;\;\;\;\;\;\;\;\;\;\;\;\;\;\;\;\;\;\;\;\;\;\;\;\;(i=1,2,\;\ldots ,\;K)\\ {\hat{x}}_{i}\;=\;\alpha_{1}x_{\left(1\right)}\;+\;\alpha_{2}x_{\left(2\right)}+\;\cdots \;+\;\;\alpha_{K}x_{\left(K\right)},\;\;\;\;\;\;(i=K+1,\;K+2,\;\;\ldots ,\;N_{T}), \end{array}$$

where the scalar weighting coefficients are normalized as

(7) $$\sum_{i=1}^{K}\alpha_{i}\;=1,\;\;\;\;\;\;\alpha_{i}\geq 0.\;\;\;\;\;\;\;\;\;\;\;\;\;\;\;\;\;\;\;\;\;\;\;\;\;\;\;\;\;\;\;\;\;\;\;\;\;\;\;\;\;\;\;\;\;\left(i=1,2,\;\ldots ,K\right).$$

In particular, we make the coefficients using random numbers as

(8) $$\alpha_{i}\;=u_{i}/\left(u_{1}\;+u_{2}\;+\cdots \;+\;u_{K}\right)$$

where $u_{i}$ is the random number with a uniform distribution over [0, 1]. Each component of the generated ${\hat{x}}_{i}$ in (6) lies between 0 and 1. The corresponding training data for the observations are as follows:

(9) $${\hat{y}}_{i}\;=\;gA{\hat{x}}_{i}\;+\;n_{i},\;\;\;\;\;\;\;\;\;\;\;\;\;\;\;\;\;\;\;\;\;\;\;\;\;\;\;\;\;\;\;\;\;\;\;(i=1,\;2,\;\ldots ,\;N_{T})$$

where $n_{i}$ is the *m*-dimensional noise vector, whose *j*-th element ${\left(n_{i}\right)}_{j}$ (*j* = 1, 2,..., *m*) is assumed to be Gaussian white noise with zero mean and variance *a*. Therefore, we generate additive noise using the random number *randn* with a standard normal distribution as follows:

(10) $${\left(n_{i}\right)}_{j}=\sqrt{a}*randn$$

#### 3.2.2. Network Architecture and Learning Procedure

**Figure 3:**
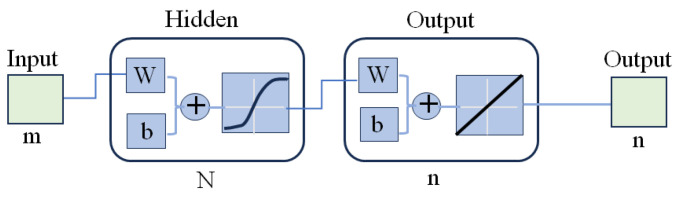
Architecture of the feedforward neural network with a structure of *m-N-n.*.

We use a simple feed-forward neural network with one hidden layer to construct a mapping from the observation space to the spectral reflectance space. Because the observation and reflectance spaces have *m-* and *n-*dimensions, respectively, the network is constructed with a structure of *m-N-n*, as shown in Figure 3, where *N* indicates the number of units in the hidden layer.

The MATLAB machine learning functions are used to construct the network [38]. Network training is performed using the following:

**net = feedforwardnet(*N*),**

**net = train(net, xdata, tdata),**

where **xdata** and **tdata** indicate the training dataset and corresponding target (output) dataset, respectively, which have the following forms:

$$\begin{array}{l} xdata=\left[xdata_{\mathrm{train}},\;xdata_{\mathrm{val}},\;xdata_{\mathrm{test}}\right],\\ tdata=\left[tdata_{\mathrm{train}},\;tdata_{\mathrm{val}},\;tdata_{\mathrm{test}}\right] \end{array}$$

The entire training data of spectral reflectances ${\hat{x}}_{i}$ $\;(i=1,\;2,\;\ldots ,\;N_{T})$ are segmented randomly into $xdata_{\mathrm{train}}$ for the network training and $xdata_{\mathrm{val}}$ for validation. In the same way, the entire training data of the corresponding observations ${\hat{y}}_{i}\;\;(i=1,\;2,\;\ldots ,\;N_{T})$ are segmented into $tdata_{\mathrm{train}}$ and $tdata_{\mathrm{val}}$. The training algorithm is based on the Levenberg–Marquardt method, and the training is iterated to reduce the MSE to an acceptable level.

The spectral reflectance corresponding to the test observation data $tdata_{\mathrm{test}}$ is predicted using the trained network, as follows:

$$x_{\mathrm{est}}=sim\left(net,\;tdata_{\mathrm{test}}\right).$$

#### 3.2.3. Determining the Optimal Reflectance Estimate

Because the augmented training data are generated using random numbers, the reflectance estimates predicted above for spectral reflectance may include outliers that differ significantly from the predictions. To improve the reliability of the reflectance estimation, the reflectance estimation is repeated, and then the median in a set of the estimated reflectances is determined as the final spectral reflectance.

$${\hat{x}}_{\mathrm{fin}}=median\left(\left\{x_{\mathrm{est}}\right\}\right)$$

Suppose we repeat the estimation process R times. Let $x_{j,\left(k\right)}$ be the estimate of the *j*-th element of the *n*-dimensional vector $x_{\mathrm{est}}$ in the *k-*th trial, arranged in ascending order as $x_{j,\left(1\right)}\leq x_{j,\left(2\right)}\leq \cdots \leq x_{j,\left(R\right)}$. The final estimate after taking the median is then described for odd and even numbers of iterations *R* as follows:

(11) x^j, fin=xj,R+12                             R: odd12xj,R2+xj,R2+1            R: even             (j=1,  2, …,  n)

**Figure 4:**
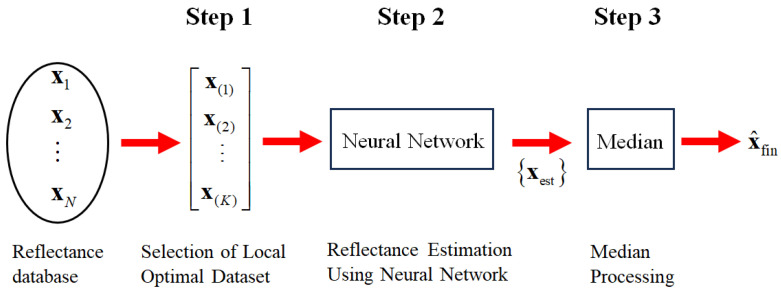
Overall flow of the proposed method for estimating spectral reflectance in three steps.

Figure 4 depicts the overall flow of the proposed method for estimating spectral reflectance in three steps.

## 4. Experimental Results

### 4.1. Experimental Setup

**Figure 5:**
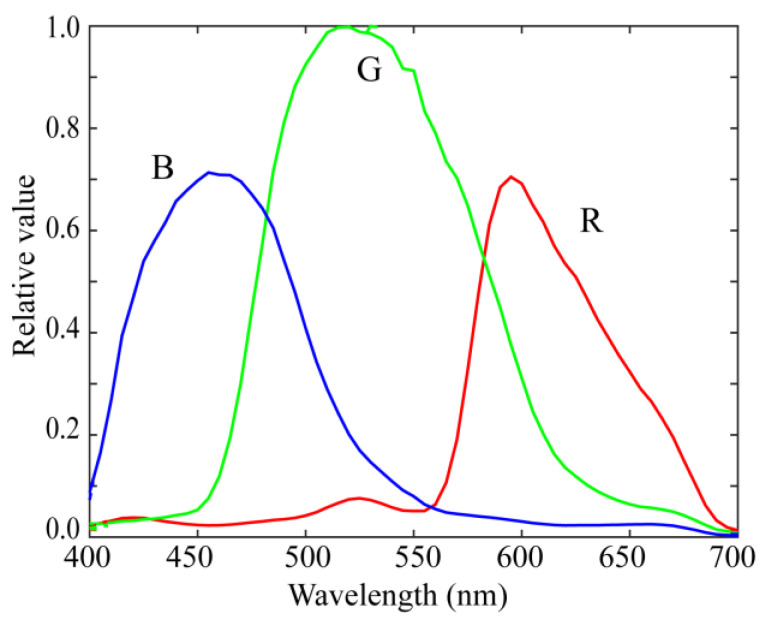
Relative RGB spectral sensitivity functions of the Apple iPhone 6s [14].

We performed experiments to validate the superiority of the proposed method for estimating surface-spectral reflectance from image data. We used a mobile phone camera, LED light sources, a standard spectral reflectance database, and standard test samples. The mobile phone camera was an Apple iPhone 6s with iOS; to further confirm the validity of the different cameras, we additionally used an Apple iPhone 8 with iOS and a Huawei P10 lite with Android OS. Figure 5 shows the relative RGB spectral sensitivity functions of the Apple iPhone 6s. The numerical data for the spectral sensitivities are available at http://ohlab.kic.ac.jp/ (accessed on 1 July 2024). Camera images were captured in a lossless raw image format in an Adobe digital negative format. The dark response was measured under dark conditions and was discarded from the camera output. The camera depth was 12 bits.

**Figure 6:**
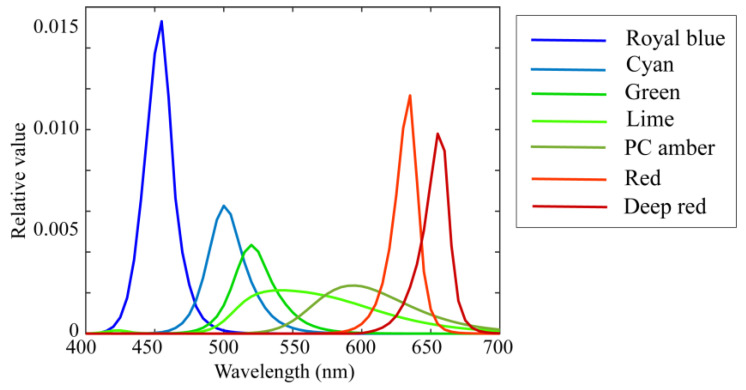
Spectral power distributions of seven LED light sources used in current experiments [14].

The illumination light sources were seven (*L* = 7) LED light sources, the spectral power distributions of which are shown in Figure 6. The standard spectral reflectance database used in the experiments is shown in Figure 2. An X-Rite Color Checker Passport Photo was used as the standard test target to validate the reflectance estimation. This target comprised 24 color checkers whose spectral reflectance values were measured using a spectral colorimeter.

Spectralon was used as a white reference standard to investigate the statistical properties of this imaging system, which was placed near the target samples. The positions are similar to those in the previous paper [13]. The parameters g and *a* of the gain and noise variance in the observation model, respectively, were determined using the calibration method in [13] based on the Spectralon data.

Since neural network processing takes a lot of time, we used a PC equipped with an NVIDIA GeForce RTX Graphics Processing Unit (GPU).

### 4.2. Bacic Performance of Proposed Method

In a previous study [14], we investigated the number *K* of local optimal reflectance candidates using different reflectance estimation methods and found that the appropriate *K* value was in the range of 5–50. Therefore, we set *K* = 25 in the current experiments and generated the training data based on the local optimal dataset $\left(x_{\left(1\right)},\;x_{\left(2\right)},\;\ldots ,\;x_{\left(25\right)}\right)$. The 600 augmented reflectance data were obtained by linear combinations of the 25 local optimal reflectances according to Equations (6)–(8). Overall, we had 625 ($N_{T}$) spectral reflectances as training data. Matrix **A** was created using the spectral sensitivity functions shown in Figure 5 and the spectral distributions of the light sources shown in Figure 6. The corresponding training data for the observation ${\hat{y}}_{i}$ were made for the respective reflectance ${\hat{x}}_{i}$ (*i* = 1,2,…, 625). The locally optimal reflectance dataset and training data were determined for each test target sample.

Our feedforward network had a structure of 21-80-61 in Figure 3, which was constructed with 21 inputs, 1 hidden layer of 80 units, and 61 outputs. The total number of reflectance and observation pairs in our dataset for each sample was 625. Of these, 550 were randomly selected for training the network, and the remaining 75 were used as validation data to investigate the proposed network method. Each pair of training data constituted the network input and output. One period in which the entire training dataset was presented was defined as an epoch. The training was iterated for as many epochs as necessary to reduce the MSE to an acceptable level. After eight epochs, the error in the validation data was sufficiently small.

The observation data for each test sample were input into the learned neural network to obtain an estimate of the spectral reflectance $x_{\mathrm{est}}$. Furthermore, to improve the estimation accuracy, we repeated the learning and testing process 10 times and finally adopted the median in the estimated reflectance set $\left\{x_{\mathrm{est}}\right\}$ as the final spectral reflectance estimate ${\hat{x}}_{\mathrm{fin}}$.

**Figure 7:**
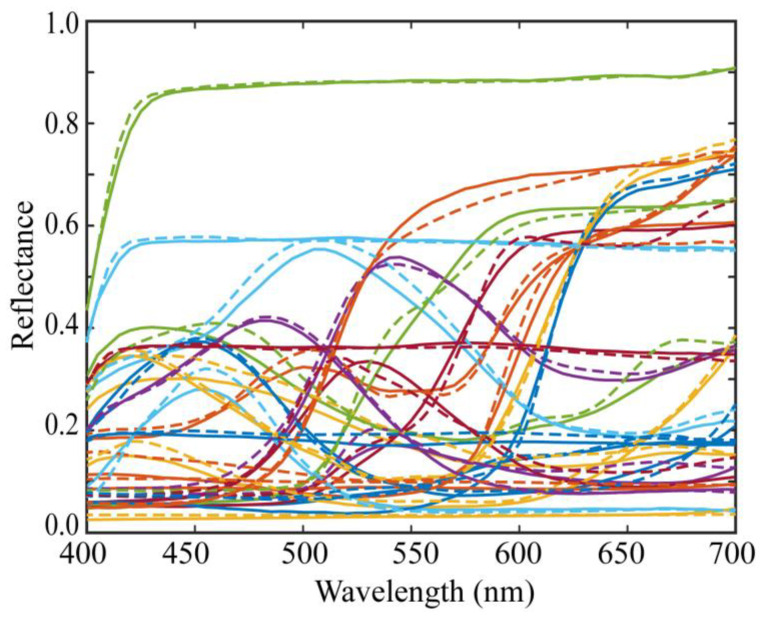
Estimation results of the spectral reflectances for the 24 color checkers when applying the proposed method to the observations using the iPhone 6s. The bold and broken curves indicate, respectively, the estimated and directly measured spectral reflectances for the 24 color checkers.

Figure 7 shows the estimation results of the above procedure for the 24 spectral reflectance of the X-Rite Color Checker. In the figure, two types of curves are compared: bold curves indicate the estimated spectral reflectances for the 24 color checkers, and broken curves indicate the directly measured spectral reflectances. The average root-mean-square error (RMSE) was calculated as the root of the average of the squared norm of the estimation error per wavelength over the 24 color checkers:

(12) $$\hat{E}[\mathrm{RMSE}]={\left\{\left(\sum_{i=1}^{24}{\left\|x_{i}-\;{\hat{x}}_{\mathrm{fin},i}\right\|}^{2}/61\right)/24\right\}}^{1/2}$$

The average RMSE was 0.0173. The estimated spectral curves in Figure 7 were smoothed using moving-average processing. However, this process hardly changed the errors.

**Figure 8:**
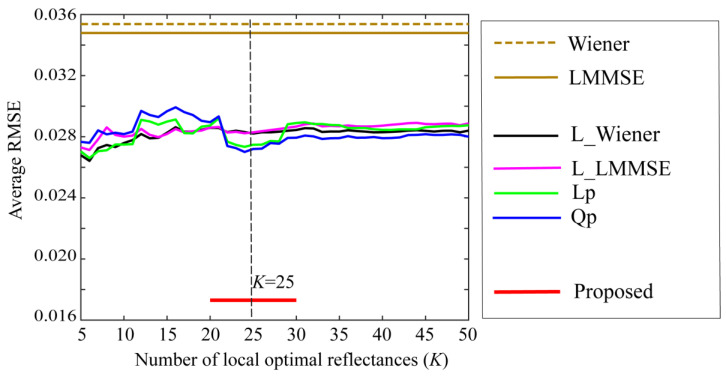
Comparison of the average RMSEs between the proposed method and the other methods. The symbols of Wiener, LMMSE, L_Wiener, L_LMMSE, Lp, and Qp represent the six estimation methods of (1) original Wiener [13], (2) original LMMSE [13], (3) local Wiener [14], (4) local LMMSE [14], (5) linear programming [14], and (6) quadratic programming [14], respectively.

Furthermore, the performance of the proposed method was compared with those of other well-known state-of-the-art methods for estimating spectral reflectance. The estimation accuracies of the six methods were investigated using the same reflectance database, camera data, and test samples described above. Figure 8 compares the average RMSEs between the proposed method and the other methods, where the symbols of Wiener, LMMSE, L_Wiener, L_LMMSE, Lp, and Qp represent the six estimation methods of (1) original Wiener, (2) original LMMSE [13], (3) local Wiener, (4) local LMMSE, (5) linear programming, and (6) quadratic programming [14], respectively. The local optimal dataset was used in Methods (3)–(6). The estimation accuracy of the proposed method is significantly superior, although the RMSEs of (3)–(6) vary slightly depending on the number of local optimal reflectance candidates *K*.

### 4.3. Effectiveness of Local Optimal Reflectance Dataset

To confirm the effectiveness of the local optimal reflectance dataset in estimating spectral reflectance, we examined several reflectance estimation methods without using the local optimal dataset and with only a neural network. All the data in the standard spectral reflectance database were used without selection.

**Figure 9:**
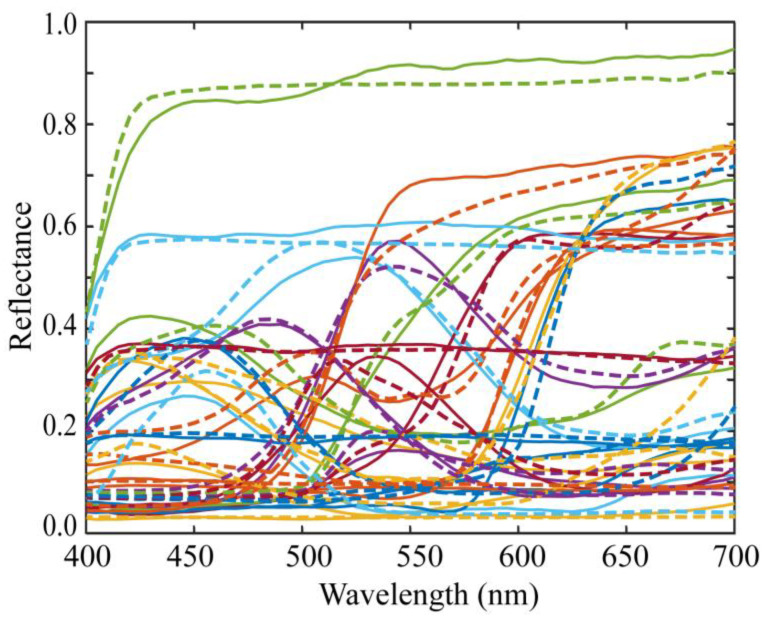
Estimation results of the spectral reflectances for the 24 color checkers when applying the network method (2) to the observations using the iPhone 6s without using the local optimal dataset. The bold and broken curves indicate, respectively, the estimated and directly measured spectral reflectances for the 24 color checkers.

First, by making the network structure multilayered and large-scale, complex mapping becomes possible to ensure that the estimation accuracy can be improved, even if learning takes a long time. Based on this idea, we constructed networks with three hidden layers with two types of structures: (1) 21-30-30-30-61 and (2) 21-30-40-50-61. The total number of training datasets was 1776; of these, 1576 reflectances were used for network training, and 200 reflectances were used for validation. The average RMSE after 10 epochs was 0.033003 and 0.0300666 for (1) and (2), respectively. Figure 9 shows the results estimated by network method (2) for the 24 spectral reflectances of the color checker. The estimation accuracy is significantly worse than the results in Section 4.2.

Next, we considered improving the estimation accuracy by increasing the amount of training data. Additional spectral reflectances were obtained by augmentation with a convex linear combination of the original reflectance data. We randomly selected 10 reflectances from the original dataset and augmented the data using a convex linear combination of these, where the weighting coefficients were normalized to satisfy Equation (7). Among the data, 4000 reflectances were used for network training, and 200 reflectances were used for validation. The network structure was 21-30-30-30-61. In this third case (3), the average RMSE after 10 epochs was 0.031144.

Thus, we see that the local optimal reflectance dataset is crucial for estimating the spectral reflectance.

### 4.4. Validity to Different Cameras

The performance evaluation described above was based on a single mobile phone camera, iPhone 6s. To further confirm the validity of the different cameras, we used an Apple iPhone 8 with iOS and Huawei P10 lite with Android OS. The spectral sensitivity function data for these cameras are available at http://ohlab.kic.ac.jp/ (accessed on 1 July 2024). The LED light sources, spectral reflectance database, and test samples of the 24 color checkers were the same as those shown in Section 4.1. Parameters *g* and *a* in the observation model were determined using the same calibration method.

**Figure 10:**
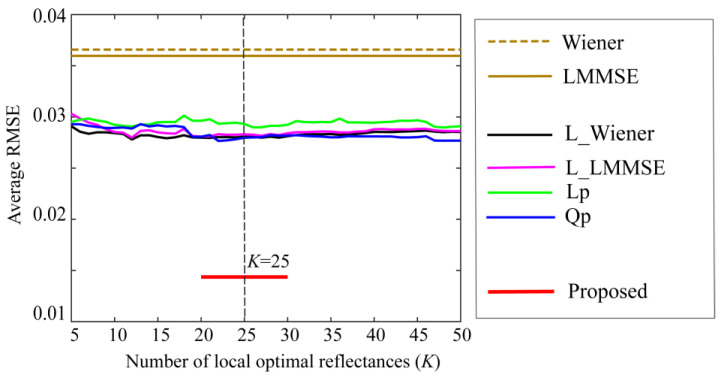
Comparison of the average RMSEs between the proposed method and the other methods when using iPhone 8.

**Figure 11:**
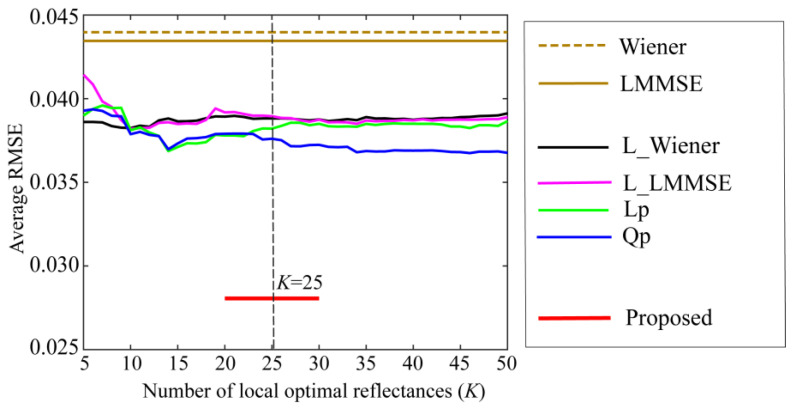
Comparison of the average RMSEs between the proposed method and the other methods when using Huawei P10 lite.

The 24 spectral reflectances of the X-Rite Color Checker were estimated based on the proposed method using observations from each camera, and the estimation accuracy was validated through comparison with other methods. The average RMSEs were 0.0144 and 0.0281 for Phone 8 and Huawei P10 lite, respectively. The estimation error increased when a Huawei camera was used. Figure 10 and Figure 11 compare the average RMSEs between the proposed method and the six other methods for the iPhone 8 and Huawei P10 lite, respectively. The estimation accuracies of the proposed method are overwhelmingly superior for both cameras, as demonstrated in the iPhone 6s.

## 5. Discussion

### 5.1. Relationship between the Statistics of Random Numbers and Estimation Accuracy

Most training data for spectral reflectance are augmented data generated by a linear combination of local optimal datasets. The weighting coefficients are calculated using random numbers. Therefore, the final spectral reflectance estimates are affected by the statistics of the random numbers used.

The augmented spectral reflectances defined in Equations (6)–(8) are rewritten as follows:

(13) $$\;\hat{x}=\sum_{i=1}^{K}\frac{u_{i}}{u_{1}\;+u_{2}\;+\cdots \;+\;u_{K}}x_{\left(i\right)},\;\;\left(i=1,2,\;\ldots ,K\right)$$

where $x_{\left(i\right)}$ are the local optimal reflectances and $u_{i}$ are independent and identically distributed random numbers with $u_{i}>0$. Let σ and $\bar{u}$ be the standard deviation and mean of $u_{i}$, respectively. The coefficient of variation *Cv* is then defined as the standard deviation divided by the mean as

(14) $$Cv=\frac{\sigma }{\bar{u}}.$$

This measure is a statistical index showing the relative variation of the random numbers.

Let us calculate *Cv* for specific distributions of random numbers.

(a) When $u_{i}$ following a uniform distribution over [0, 1], $Cv=\frac{1}{\sqrt{3}}$.

(b) When $u_{i}$ following a chi-square ($\chi^{2}$) distribution with one degree of freedom,

$Cv=\sqrt{2}$.

In case (b), the variation is $\sqrt{6}$ times larger than that in case (a).

Because the coefficient of variation differs depending on the distribution of the random numbers, it is likely to affect the final estimation accuracy of the spectral reflectance. Therefore, in addition to the above experiments using uniform random numbers, we conducted experiments on spectral reflectance estimation using random numbers with a chi-square distribution and compared the estimation accuracies in both cases. Table 1 compares the average RMSEs of the reflectance estimation using random numbers with two different distributions and three different cameras. The training data for spectral reflectance based on uniform random numbers with smaller *Cv* are superior in terms of estimation accuracy.

### 5.2. Evaluation by Color Difference

In this study, RMSE is mainly used for evaluating the accuracy of reflectance estimation. To make clear to what extent the deviation in the estimated reflectances is visible to the human eye, color difference rather than the RMSE of spectral difference may be useful. Therefore, we calculated CIEDE2000 values between the estimated reflectances and directly measured spectral reflectances for the 24 color checkers.

Table 2 compares the average RMSEs and the (average, median, 90% percentile) CIEDE2000 values between the three different methods of the original LMMSE [13], the quadratic programming using local optimal reflectance dataset [14], and the proposed method. The RMSEs were computed based on the spectral differences between estimated reflectances and directly measured spectral reflectances for the 24 color checkers, and the CIE DE2000s were calculated based on the color differences under CIE D65 light between estimated reflectances and directly measured spectral reflectances for the 24 color checkers.

## 6. Conclusions

In this study, we have proposed a novel method that combines model-based and training-based approaches to improve the estimation accuracy of surface-spectral reflectance from the camera response to an object surface. A multispectral image acquisition system was modeled in the visible wavelength range using three spectral functions: the spectral sensitivities of an RGB camera, spectral power distributions of multiple LED light sources, and unknown surface-spectral reflectance. Camera response was described as a generalized linear model that includes additive noise and a gain parameter.

The proposed method comprised two main stages: the first stage was based on the model base, where the local optimal reflectance dataset was selected as the most reliable candidate set from a standard reflectance database, and the second stage was based on the training-based method, where the best estimate was determined by applying a neural network method to the selected local optimal dataset only.

In the first stage, the camera response observations were predicted for the respective reflectances in the database, and the local optimal candidates were selected in the order of the lowest error between the real observation and prediction. In the second stage, the training data for spectral reflectances consisted of the original locally optimal dataset and augmented data generated by a convex linear combination of this dataset, with weighting coefficients derived from random numbers. A simple feedforward neural network with one hidden layer was used to construct the mapping from the low-dimensional observation space using the camera response to the high-dimensional spectral reflectance space.

The neural network was trained to minimize MSE. To further improve the reliability of the estimate, the reflectance estimation was repeated, and the median in a set of estimated reflectances was determined as the final spectral reflectance.

Experiments were conducted using 3 mobile phone cameras, 7 LED light sources, a standard spectral reflectance database with 1776 reflectances, and 24 color checkers. The performance of the proposed method was examined in detail. We investigated estimation methods based on a neural network using the entire database without selection and confirmed the effectiveness of the local optimal reflectance dataset. We demonstrated that the estimation accuracies of the proposed method exceed those of the other methods. Furthermore, we discussed the statistics of the random numbers used, which affects the estimation accuracy.

The strength of the proposed method is its outstanding estimation accuracy. However, the computation time for the current approach is long. When using a laptop computer equipped with an NVIDIA GeForce RTX GPU, it took about 25 h to obtain the estimation results for 24 color checkers after repetition of the learning and testing process 10 times. A key challenge for the future is reducing the computation time required for obtaining estimation results, which is heavily dependent on the processing speed of the computer used.

## Figures and Tables

**Table 1:** Comparison of the average RMSEs in reflectance estimation when using random numbers with two different distributions and using three different cameras.

	iPhone 6s	iPhone 8	Huawei P10 Lite
Uniform distribution	0.0173	0.0144	0.0281
Chi-square distribution	0.0195	0.0158	0.0296

**Table 2:** Comparison of the average RMSE and the (average, median, 90% percentile) CIEDE2000 values between the three different methods of the original LMMSE, the quadratic programming, and the proposed method.

	RMSE Average	CIEDE2000 Average	CIEDE2000 Median	CIEDE2000 90% Percentile
Original LMMSE [13]	0.0347	2.756	3.090	3.726
Quadratic programming [14]	0.0272	1.576	1.060	3.653
Proposed method	0.0173	1.087	0.0677	2.790

## Data Availability

Data underlying the results presented in this paper are available at http://ohlab.kic.ac.jp/ (accessed on 1 August 2024).
